# The impact of the surgical Apgar score on oncological outcomes in patients with colorectal cancer: a propensity score-matched study

**DOI:** 10.1186/s12957-022-02545-x

**Published:** 2022-03-10

**Authors:** Atsushi Sugimoto, Tatsunari Fukuoka, Hisashi Nagahara, Masatsune Shibutani, Yasuhito Iseki, Maho Sasaki, Yuki Okazaki, Kiyoshi Maeda, Masaichi Ohira

**Affiliations:** 1 Department of Gastroenterological Surgery, Osaka City University Graduate School of Medicine, 1-4-3 Asahimachi, Abeno-ku, Osaka, 545-8585 Japan; 2 Department of Gastroenterological Surgery, Osaka City General Hospital, 2-13-22 Miyakojimahondori, Miyakojima-ku, Osaka, 534-0021 Japan

**Keywords:** Surgical Apgar score, Colorectal cancer, Postoperative complications, Prognosis, Cancer-specific survival

## Abstract

**Background:**

The surgical Apgar score (SAS) predicts postoperative complications (POCs) following gastrointestinal surgery. Recently, the SAS was reported to be a predictor of not only POCs but also prognosis. However, the impact of the SAS on oncological outcomes in patients with colorectal cancer (CRC) has not been fully examined. The present study therefore explored the oncological significance of the SAS in patients with CRC, using a propensity score matching (PSM) method.

**Methods:**

We retrospectively analyzed 639 patients who underwent radical surgery for CRC. The SAS was calculated based on three intraoperative parameters: estimated blood loss, lowest mean arterial pressure, and lowest heart rate. All patients were classified into 2 groups based on the SAS (≤6 and >6). The association of the SAS with the recurrence-free survival (RFS), overall survival (OS), and cancer-specific survival (CSS) was analyzed.

**Results:**

After PSM, each group included 156 patients. Univariate analyses revealed that a lower SAS (≤6) was significantly associated with a worse OS and CSS. A multivariate analysis revealed that the age ≥75 years old, ASA-Physical Status ≥3, SAS ≤6, histologically undifferentiated tumor type, and an advanced pStage were independent factors for the OS, and open surgery, a SAS ≤6, histologically undifferentiated tumor type and advanced pStage were independent factors for the CSS.

**Conclusions:**

A lower SAS (≤6) was an independent prognostic factor for not only the OS but also the CSS in patients with CRC, suggesting that the SAS might be a useful biomarker predicting oncological outcomes in patients with CRC.

## Introduction

Colorectal cancer (CRC) was estimated to account for more than 1.9 million new colorectal cancer cases and 935,000 deaths in 2020, ranking third in terms of incidence but second in terms of mortality globally [1]. Although surgical resection is the standard treatment for local and regional CRC worldwide, the mortality from CRC remains unsatisfactory.

Notably, among patients who undergo curative surgery for CRC, approximately one third will develop disease recurrence, underscoring the importance of developing biomarkers to identify patients who may require postoperative intensification of treatment [2]. Postoperative complications (POCs) are reportedly significantly associated with a poor prognosis in CRC [3]. Therefore, predicting and preventing POCs might be one way to increase the survival in CRC.

The surgical Apgar score (SAS) system was developed by Gawande et al. to predict POCs in general surgery in 2007 [4]. The SAS consists of three intraoperative parameters: the estimated blood loss (EBL), the lowest mean arterial pressure (LMAP), and the lowest heart rate (LHR). The SAS has been validated as a predictor of POCs in CRC surgeries [5]. Previously, we reported that the SAS was a valuable predictor of severe complications after CRC surgery in elderly patients [6]. One of the reasons why the SAS is able to predict POCs is that it reflects the intraoperative hemodynamic stability in patients with gastrointestinal cancer. Recent studies have highlighted the significant impact of the SAS on not only POCs but also the overall survival (OS) in gastrointestinal cancer [7, 8]. However, the impact of the SAS on oncological outcomes in patients with CRC has not been fully examined.

We hypothesized that the SAS, which reflects intraoperative hemodynamics, would affect not only the OS but also the oncological long-term outcomes, such as the recurrence-free survival (RFS) and cancer-specific survival (CSS), in CRC patients. The present study therefore assessed the impact of the SAS on oncological outcomes after radical surgery in CRC patients, using a propensity score matching (PSM) method.

## Materials and methods

### Patients

We retrospectively analyzed consecutive patients who underwent radical surgery under general anesthesia for CRC at the Department of Gastroenterological Surgery, Osaka City University Hospital, from January 2008 to December 2014. We excluded patients with pathological Stage 0 or IV, non-curative (R1 or R2) resection, preoperative treatment (chemotherapy and/or radiotherapy), synchronous surgeries for other cancers, and histologically atypical tumors, such as squamous cell carcinoma, small-cell carcinoma, gastrointestinal stromal tumor (GIST), or melanoma. The following clinical and surgical data were collected from electronic medical records: age, gender, body mass index (BMI), the presence of current smoking, serum albumin level, serum C-reactive protein (CRP) level, the Glasgow prognostic score (GPS) [9], the American Society of Anesthesiologists classification of physical status (ASA-PS), tumor location (colon and rectum), pathological T (pT) stage, pathological N (pN) stage, pathological TNM stage (pStage), histological tumor type (differentiated type; well- or moderately differentiated adenocarcinoma and undifferentiated type; poorly differentiated and mucinous adenocarcinoma), operative procedure (laparoscopy and open surgery), operation time, intraoperative EBL, transfusion, intraoperative LMAP, and intraoperative LHR. Comorbidities were evaluated according to the Charlson Comorbidity Index (CCI) [10]. The pathological TNM stage was determined based on the 8th edition of the Union for International Cancer Control TNM classification of malignant tumors [11].

### SAS

We used the original the SAS scoring system to calculate the SAS [4]. The three intraoperative SAS parameters (EBL, LMAP, and LHR) were extracted from electronic anesthesia records. The score is the sum of the points from each category (Table 1). The cut-off value of the SAS was determined as the point on the receiver operating characteristic (ROC) curve predicting severe POCs, defined as grade ≥III according to the Clavien-Dindo classification (CDC) [12], at which the Youden index was maximal. All patients were classified into one of two groups based on this cut-off value.

**Table 1:** The surgical Apgar score

	0 point	1 point	2 points	3 points	4 points
Estimated blood loss (mL)	> 1000	601–1000	101–600	≤ 100	-
Lowest mean arterial pressure (mmHg)	< 40	40–54	55–69	≥ 70	-
Lowest heart rate (beats/min)	> 85	76–85	66–75	56–65	≤ 55

### Treatment strategy

Our treatment strategy for CRC is based on the Japanese Society for Cancer of the Colon and Rectum (JSCCR) guidelines [13]. All patients underwent various radiological tests for the preoperative diagnosis and staging, such as colonoscopy and contrast-enhanced computed tomography (CT). Radical surgery was defined as no residual tumor cells microscopically at the stump of the surgical specimen with an adequate surgical margin. General anesthesia was mainly performed by intravenous anesthesia, and the anesthesiologists were involved in the anesthesia management of all cases. Adjuvant chemotherapy was performed for patients with pathological stage II/III disease. Patients received monotherapy using an oral pro-drug based on 5-FU, such as capecitabine or combination therapy with 5-FU and oxaliplatin, such as 5-fluorouracil/leucovorin plus oxaliplatin (FOLFOX) or capecitabine plus oxaliplatin (CapeOX).

### POCs and the prognosis

Severe POCs were defined as grade ≥III according to the CDC that developed within 30 days after surgery. The prognosis was analyzed based on the information in the electronic medical record. Patients were followed-up at intervals of three to 6 months until the end of this study or death. The OS, RFS, and CSS were calculated from the start date of the operation to the date of last follow-up or death, to the confirmed date of recurrence or death and to the date of last follow-up or death due to CRC, respectively.

### Statistical analyses

Data of continuous variables are presented as median (interquartile range [IQR]). The cutoff value of the SAS was calculated by the ROC curve for severe POCs. The PSM was performed for minimizing confounding based on clinicopathological characteristics including age, sex, gender, BMI, smoking, CCI, serum albumin level, serum CRP level, ASA-PS, tumor location (colon or rectum), pT, pN, pStage, histological tumor type (differentiated type or undifferentiated type), operative procedure (laparoscopy or open surgery), and operative time. Patients were matched 1:1 by the neighbor matching method. The univariate analysis was performed by the Mann-Whitney *U* test for continuous variables and by the chi-squared test for categorical variables. Survival probabilities (OS, RFS, and CSS) were calculated by Kaplan–Meier survival curves and statistically compared by the log-rank test. Univariate and multivariate analyses using the Cox proportional hazard model were performed to identify significant prognostic factors for OS and CSS. Hazard ratios (HRs) and 95% confidence intervals (CIs) were calculated. Values of *p*<0.05 were considered significant. All data analyses were conducted using the JMP® 13 software program (SAS Institute Inc., Cary, NC, USA).

### Ethics

The Ethics Committee at Osaka City University approved this retrospective study of clinical data, which was conducted in accordance with the principles of the Declaration of Helsinki.

## Results

### Patients’ characteristics

A total of 639 (colon cancer in 460 cases and rectal cancer in 179 cases) patients were enrolled in this study. Severe POCs of CDC grade ≥III were observed in 102 patients (16.0%). According to the ROC curve analysis, patients were divided into two groups based on the cutoff value of the SAS. Before PSM, the patients with SAS ≤6 (*n*=190, 29.7%) were assigned to the low-SAS group, and those with SAS ≥7 (*n*=449, 70.3%) were assigned to the high-SAS gr oup. After PMS, each group included 156 patients.

### Clinicopathological characteristics of the high- and low-SAS groups

Before PMS, the low-SAS group more frequently included patients with GPS ≥1 (*p*<0.001), advanced pT (*p*=0.003), advanced pStage (*p*=0.005), histologically undifferentiated tumor type (*p*=0.03), open surgery (*p*<0.001), larger EBL (*p*<0.001), longer operative time (*p*=0.023), and transfusion (*p*<0.001) than the high-SAS group (Table 2). After PMS, the clinicopathological characteristics were well balanced. The low-SAS group more frequently included patients with larger EBL (*p*<0.001) than the high-SAS group (Table 2).

**Table 2:** Clinicopathological characteristics of the high- and low-SAS groups before and after propensity score matching

Characteristics	Before matching, *n* (%)		*P* value	After matching, *n* (%)		*P* value
	Group H (SAS >6)	Group L (SAS ≤6)		Group H (SAS >6)	Group L (SAS ≤6)	
	*n* = 449	*n* = 190		*n* = 156	*n* = 156	
Age						
Years, IQR	70 (62−76)	69 (62−75)	0.762	69 (61−76)	69 (62−75)	0.82
Gender						
Female	189 (68.7%)	86 (31.3%)	0.46	70 (50.4%)	69 (49.6%)	0.909
Male	260 (71.4%)	104 (28.6%)		86 (49.7%)	87 (50.3%)	
BMI						
kg/m^2^, IQR	22.4 (20.4−24.4)	21.8 (19.9−24.2)	0.167	22.3 (20.6−23.9)	22.0 (19.8−24.3)	0.453
Smoking						
Yes	167 (71.1%)	68 (28.9%)	0.737	54 (48.2%)	58 (51.8%)	0.637
No	282 (69.8%)	122 (30.2%)		102 (51.0%)	98 (49.0%)	
CCI						
< 1	231 (69.0%)	104 (31.0%)	0.447	77 (47.2%)	86 (52.8%)	0.308
≥ 1	218 (71.7%)	86 (28.3%)		79 (53.0%)	70 (47.0%)	
Albumin						
g/dL	4.1 (3.8−4.3)	4.0 (3.7−4.3)	0.003	4.1 (3.7−4.3)	4.1 (3.8−4.3)	0.985
CRP						
mg/dL	0.08 (0.03−0.29)	0.16 (0.05−0.61)	<0.001	0.09 (0.03−0.5)	0.14 (0.04−0.36)	0.18
GPS						
0	394 (73.5%)	142 (26.5%)	<0.001	132 (50.4%)	130 (49.6%)	0.758
1,2	55 (53.4%)	48 (46.6%)		24 (48.0%)	26 (52.0%)	
ASA-PS						
1	71 (70.3%)	30 (29.7%)	0.8	24 (53.3%)	21 (46.7%)	0.862
2	325 (70.8%)	134 (29.2%)		114 (49.1%)	118 (50.9%)	
3	53 (67.1%)	26 (32.9%)		18 (51.4%)	17 (48.6%)	
Location						
Colon	329 (71.5%)	131 (28.5%)	0.266	111 (49.1%)	115 (50.9%)	0.612
Rectum	120 (67.0%)	59 (33.0%)		45 (52.3%)	41 (47.7%)	
pT						
1	132 (79.5%)	34 (20.5%)	0.003	38 (52.8%)	34 (47.2%)	0.469
2	76 (75.2%)	25 (24.8%)		23 (53.5%)	20 (46.5%)	
3	168 (65.9%)	87 (38.7%)		70 (51.5%)	66 (48.5%)	
4	73 (62.4%)	44 (37.6%)		25 (41.0%)	36 (59.0%)	
pN						
0	326 (72.3%)	125 (27.7%)	0.064	106 (50.0%)	106 (50.0%)	1
1	89 (69.0%)	40 (31.0%)		32 (50.0%)	32 (50.0%)	
2	34 (57.6%)	25 (42.4%)		18 (50.0%)	18 (50.0%)	
pStage						
I	186 (77.8%)	53 (22.2%)	0.005	55 (52.9%)	49 (47.1%)	0.712
II	140 (66.0%)	72 (34.0%)		51 (47.2%)	57 (52.8%)	
III	123 (65.4%)	65 (34.6%)		50 (50.0%)	50 (50.0%)	
Histologically tumor type						
Differentiated	432 (71.2%)	175 (28.8%)	0.03	146 (50.0%)	146 (50.0%)	1
Undifferentiated	17 (53.1%)	15 (46.9%)		10 (50.0%)	10 (50.0%)	
Procedures						
Laparoscopy	348 (81.1%)	81 (18.9%)	<0.001	81 (50.0%)	81 (50.0%)	1
Open surgery	101 (48.1%)	109 (51.9%)		75 (50.0%)	75 (50.0%)	
EBL						
mL, IQR	38 (20−80)	168 (50−410)	<0.001	50 (20−100)	118 (31−286)	<0.001
Operative time						
min, IQR	211 (175−266)	234 (176−287)	0.023	213 (178−270)	226 (170−266)	0.798
Transfusion						
Yes	19 (40.4%)	28 (59.6%)	<0.001	8 (34.8%)	15 (65.2%)	0.129
No	430 (72.6%)	162 (27.4%)		148 (51.2%)	141 (48.8%)	
POC						
≤ CDC II	393 (73.2%)	144 (26.8%)	<0.001	135 (52.3%)	123 (47.7%)	0.073
≥ CDC III	56 (54.9%)	46 (45.1%)		21 (38.9%)	33 (61.1%)	
Postoperative stay						
Days, IQR	12 (10−18)	14 (11−23)	<0.001	13 (11−19)	13 (10−22)	0.592

*IQR* Interquartile range, *BMI* Body mass index, *CCI* Charlson Comorbidity Index, *CRP* C-reactive protein, *GPS* Glasgow prognostic score, *POC* Postoperative complication, *CDC* Clavien-Dindo classification

### Postoperative outcomes

Before PMS, the low-SAS group more frequently included patients with severe POCs (CDC grade ≥III) (*p*<0.001) and who had a significantly longer postoperative stay (*p*<0.001) than the high-SAS group (Table 2). After PMS, severe POCs and postoperative stay were not significantly different between the two groups.

### The prognosis

**Fig. 1:**
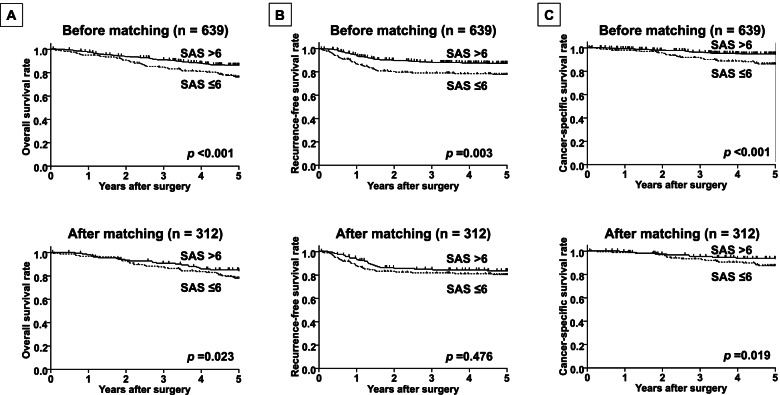
The prognosis based on the surgical Apgar score (SAS) before and after propensity score matching. **A** The overall survival (OS). The 5-year OS rates in the low-SAS group (≤6) were significantly lower than those in the high-SAS group (≥7) (*p* <0.001). **B** The recurrence-free survival (RFS). The 5-year RFS rates in the low-SAS group (≤6) were significantly lower than those in the high-SAS group (≥7) (*p*=0.003). **C** The cancer-specific survival (CSS). The 5-year CSS rates in the low-SAS group (≤6) were significantly lower than those in the high-SAS group (≥7) (Kaplan-Meier; *p* <0.001)

The median follow-up time was 63.4 (IQR, 54.8−83.0) months for all patients. Before PMS, recurrences were observed in 96 cases (15.0%). Deaths due to CRC were observed in 61 cases (9.5%). A total of 142 deaths (22.2%) were observed. The 5-year OS, RFS, and CSS rates for the entire study population were 82.4%, 86.1%, and 91.8%, respectively. After PMS, recurrences were observed in 56 cases (17.9%). Deaths due to CRC were observed in 38 cases (12.2%). A total of 75 deaths (24.0%) were observed. The 5-year OS, RFS, and CSS rates for the matched patients were 81.3%, 84.1%, and 90.0%, respectively. Kaplan-Meier survival curves comparing the OS, RFS, and CSS between the two groups are shown in Fig. 1A–C. Before PMS, the OS, RFS, and CSS rates in the low-SAS group were significantly lower than those in the high-SAS group (*p*<0.001, *p*=0.003, and *p*<0.001, respectively). After PMS, the OS and CSS rates in the low-SAS group were significantly lower than those in the high-SAS group (*p*=0.023 and *p*=0.019, respectively).

### Univariate and multivariate analyses for the OS and CSS

The results of univariate and multivariate analyses for the OS and CSS before and after PMS are summarized in Table 3. Before PMS, in the univariate analyses for the OS, age ≥75 years old, CCI ≥1, GPS ≥1, ASA-PS ≥3, open surgery, SAS ≤6, histologically undifferentiated tumor type, pStage III, and severe POCs were significantly associated with a worse OS. In the multivariate analysis for the OS using variables with *p*<0.1 in univariate analyses, age ≥75 years old, CCI ≥1, ASA-PS ≥3, SAS ≤6, histologically undifferentiated tumor type, and pStage III were identified as independent prognostic factors for the OS. In contrast, in the univariate analyses for the CSS, open surgery, SAS ≤6, rectal cancer, histologically undifferentiated tumor type, pStage III, severe POCs, and adjuvant chemotherapy were significantly associated with a worse CSS. In the multivariate analysis for the CSS using variables with *p*<0.1 in univariate analyses, SAS ≤6 and pStage III were identified as independent prognostic factors for the CSS. After PMS, in the univariate analyses for the OS, age ≥75 years old, CCI ≥1, ASA-PS ≥3, open surgery, SAS ≤6, histologically undifferentiated tumor type, and pStage III were significantly associated with a worse OS. In the multivariate analysis for the OS using variables with *p*<0.1 in univariate analyses, age ≥75 years old, ASA-PS ≥3, SAS ≤6, histologically undifferentiated tumor type, and pStage III were identified as independent prognostic factors for the OS. In contrast, in the univariate analyses for the CSS, open surgery, SAS ≤6, histologically undifferentiated tumor type, pStage III, and adjuvant chemotherapy were significantly associated with a worse CSS. In the multivariate analysis for the CSS using variables with *p*<0.1 in univariate analyses, open surgery, SAS ≤6, histologically undifferentiated tumor type, and pStage III were identified as independent prognostic factors for the CSS.

**Table 3:** Results of univariate and multivariate analyses for the OS and CSS before and after propensity score matching

	Analysis for OS (before matching)				Analysis for OS (after matching)			
	Univariate analysis		Multivariate analysis		Univariate analysis		Multivariate analysis	
	HR (95% CI)	*p* value	HR (95% CI)	*p* value	HR (95% CI)	*p* value	HR (95% CI)	*p* value
Age ≥75 vs <75 (years old)	2.67 (1.90−3.74)	<0.001	2.55 (1.79−3.64)	<0.001	2.50 (1.57−3.96)	<0.001	2.59 (1.59−4.19)	<0.001
Male vs female	1.30 (0.93−1.85)	0.126			1.06 (0.67−1.69)	0.804		
BMI ≥25 vs <25 (kg/m^2)^	0.90 (0.58−1.36)	0.626			1.04 (0.57−1.79)	0.889		
CCI ≥1 vs CCI 0	1.99 (1.42−2.81)	<0.001	1.58 (1.09−2.30)	0.015	1.58 (1.00−2.52)	0.049	1.32 (0.81−2.18)	0.27
GPS ≥1 vs GPS 0	2.18 (1.47−3.14)	<0.001	1.29 (0.85−1.92)	0.227	1.44 (0.80−2.44)	0.216		
ASA-PS 3 vs ASA-PS 1, 2	4.09 (2.81−5.85)	<0.001	3.00 (1.99−4.49)	<0.001	3.15 (1.80−5.25)	<0.001	2.58 (1.41−4.53)	0.003
Open surgery vs laparoscopy	1.88 (1.34−2.62)	<0.001	1.30 (0.90−1.88)	0.164	1.83 (1.15−2.94)	0.01	1.58 (0.99−2.56)	0.057
Operative time ≥218 vs <218 (min)	1.02 (0.73−1.43)	0.901			0.73 (0.46−1.14)	0.166		
Transfusion	1.64 (0.94−2.68)	0.08	0.61 (0.34−1.05)	0.075	1.23 (0.51−2.51)	0.609		
SAS ≤6 vs >6	1.81 (1.29−2.53)	<0.001	1.51 (1.04−2.17)	0.03	1.71 (1.08−2.77)	0.022	1.76 (1.11−2.89)	0.016
Rectal cancer vs colon cancer	1.09 (0.75−1.55)	0.653			1.00 (0.59−1.62)	0.998		
Undifferentiated type vs differentiated type	3.17 (1.80−5.19)	<0.001	2.86 (1.58−4.86)	<0.001	3.32 (1.59−6.20)	0.002	3.33 (1.55−6.50)	0.003
pStage III vs pStage I and II	1.78 (1.26−2.49)	0.001	1.90 (1.33−2.69)	<0.001	1.93 (1.21−3.03)	0.006	2.27 (1.40−3.66)	0.001
POC ≥CDC III vs ≤CDC II	1.72 (1.15−2.51)	0.01	1.51 (0.99−2.25)	0.057	1.63 (0.93−2.71)	0.086	1.19 (0.66−2.06)	0.551
Adjuvant chemotherapy	0.86 (0.60−1.21)	0.39			0.91 (0.56−1.44)	0.39		
	Analysis for CSS (before matching)				Analysis for CSS (after matching)			
	Univariate analysis		Multivariate analysis		Univariate analysis		Multivariate analysis	
	HR (95% CI)	*p* value	HR (95% CI)	*p* value	HR (95% CI)	*p* value	HR (95% CI)	*p* value
Age ≥75 vs <75 (years old)	1.58 (0.91−2.66)	0.101			1.67 (0.83−3.24)	0.148		
Male vs Female	0.80 (0.48−1.32)	0.381			0.57 (0.29−1.07)	0.082	0.60 (0.30−1.16)	0.126
BMI ≥25 vs <25 (kg/m^2)^	1.50 (0.83−2.57)	0.173			1.71 (0.81−3.37)	0.15		
CCI ≥1 vs CCI 0	1.00 (0.60−1.65)	0.998			0.85 (0.44−1.60)	0.608		
GPS ≥1 vs GPS 0	1.78 (0.94−3.15)	0.074	1.16 (0.59−2.17)	0.649	0.99 (0.37−2.21)	0.98		
ASA-PS 3 vs ASA-PS 1, 2	1.89 (0.90−3.56)	0.088	1.94 (0.90−3.77)	0.086	1.85 (0.70−4.14)	0.199		
Open surgery vs laparoscopy	2.35 (1.42−3.90)	0.001	1.45 (0.82−2.57)	0.199	1.96 (1.03−3.89)	0.041	2.12 (1.10−4.26)	0.025
Operative time ≥218 vs <218 (min)	1.45 (0.87−2.45)	0.157			0.90 (0.48−1.73)	0.754		
Transfusion	1.65 (0.68−3.39)	0.243			1.45 (0.43−3.64)	0.506		
SAS ≤6 vs >6	2.64 (1.60−4.39)	<0.001	1.88 (1.07−3.30)	0.028	2.23 (1.15−4.58)	0.017	2.63 (1.34−5.48)	0.005
Rectal cancer vs colon cancer	1.89 (1.13−3.13)	0.016	1.44 (0.82−2.48)	0.199	1.38 (0.69−2.64)	0.349		
Undifferentiated type vs differentiated type	4.85 (2.30−9.22)	<0.001	2.06 (0.94−4.12)	0.069	4.78 (1.90−10.4)	0.002	3.80 (1.50−8.41)	0.007
pStage III vs pStage I and II	5.84 (3.45−10.3)	<0.001	5.85 (2.88−12.1)	<0.001	6.00 (3.05−12.6)	<0.001	6.62 (2.70−17.1)	<0.001
POC ≥CDC III vs ≤CDC II	1.97 (1.08−3.41)	0.029	1.67 (0.90−2.96)	0.104	1.63 (0.72−3.31)	0.225		
Adjuvant chemotherapy	3.10 (1.86−5.31)	<0.001	0.83 (0.42−1.71)	0.612	2.79 (1.45−5.63)	0.002	0.73 (0.31−1.80)	0.493

*OS* Overall survival, *CSS* Cancer-specific survival, *BMI* Body mass index, *CCI* Charlson comorbidity index, *GPS* Glasgow prognostic score, *POC* Postoperative complication, *CDC* Clavien-Dindo classification

### Subgroup analyses

**Fig. 2:**
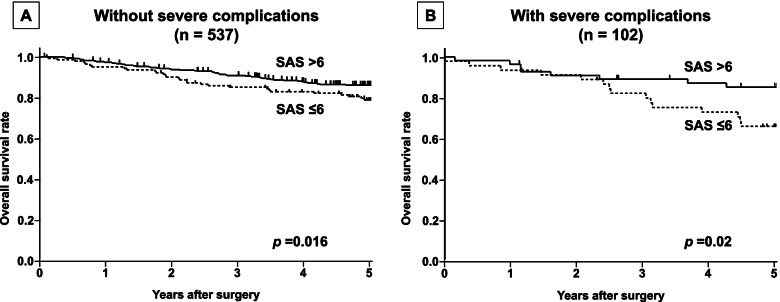
The overall survival (OS) in patients with or without severe complications. **A** The OS in 537 patients without severe complications. The OS rates in the low-SAS group (≤6) were significantly lower than that in the high-SAS group (≥7) (*p*=0.016). **B** The OS in 102 patients with severe complications. The OS rates in the low-SAS group (≤6) were significantly lower than that in the high-SAS group (≥7) (*p*=0.02)

**Fig. 3:**
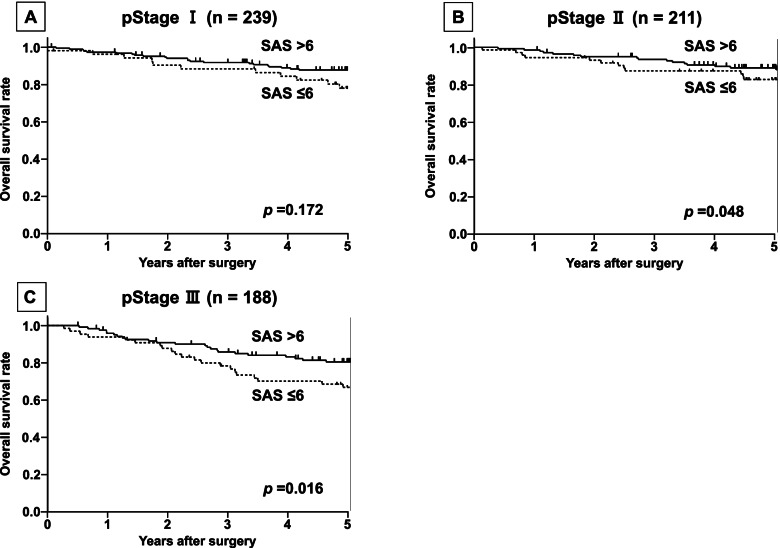
The overall survival (OS) according to pStage. **A** The OS in 239 patients with pStage I colorectal cancer. The OS rates have no significant difference between low SAS (≤6) and high SAS (≥7) (*p* =0.172). **B** The OS in 211 patients with pStage II colorectal cancer. The OS rates in the low-SAS group (≤6) were significantly lower than that in the high-SAS group (≥7) (*p* =0.048). **C** The OS in 188 patients with pStage III colorectal cancer. The OS rate in the low-SAS group (≤6) was significantly lower than that in the high-SAS group (≥7) (*p* =0.016)

A subgroup analysis according to the presence of severe POCs was conducted. The Kaplan-Meier survival curves comparing the OS based on the SAS in patients with and without severe POCs are shown in Fig. 2A, B. The OS rates in the low-SAS group were significantly lower than those in the high-SAS group among the patients with and without severe POCs (*p*=0.02 and *p*=0.016, respectively). A subgroup analysis according to the pStage (I, II, and III) was also conducted. The Kaplan-Meier survival curves comparing the OS based on the SAS in patients with pStage I, II, and III diseases are shown in Fig. 3A−C. The OS rates in the low-SAS group were significantly lower than those in the high-SAS group among patients with pStage II and III diseases (*p*=0.048 and *p*=0.016, respectively), while no significant difference was seen among the patients with pStage I disease (*p*=0.172).

## Discussion

In this study, we evaluated the SAS in patients who underwent radical surgery for CRC, before and after PMS. We identified a lower SAS (≤6) as an independent prognostic factor for the OS and CSS. Nakagawa et al. previously reported that the SAS predicted not only POCs but also the OS in esophageal cancer patients [7], and Yamada et al. reported that the SAS predicted the OS in gastric cancer patients [8]. However, the association between the SAS and oncological outcomes in CRC patients has been unclear. To our knowledge, this is the first study to clarify the impact of the SAS on the RFS and CSS in CRC patients. Our results suggested that the SAS might be a useful biomarker predicting oncological outcomes after radical surgery in CRC patients.

In this study, an older age (≥75), a higher ASA-PS (≥3), a lower SAS (≤6), histologically undifferentiated tumor type, and advanced pStage (≥III) were identified as independent factors for the OS after PMS. Our results were consistent with those of previous studies [14–16]. However, the impact of SAS on the OS has not been fully examined in CRC. An explanation concerning the correlation of the SAS with the OS has been considered. First, the SAS, consists of EBL, LMAP, and LHR, reflects intraoperative hemodynamics. Previous studies reported that significant blood loss, intraoperative hypotension, and a higher heart rate were associated with a poor prognosis in CRC [17–19]. These studies further indicated that hemodynamic instability might affect the survival in CRC. Second, the SAS reflects surgical stress, as significant blood loss, a large incision, and prolonged operation time result in a low SAS. In the present study, a lower SAS was more frequent in patients with more blood loss, open surgery, and a longer operation time. Our results were consistent with those of the previous study [20]. Finally, a low SAS was associated with POCs. POCs affect the prognosis in CRC because of marked postoperative inflammation and a poor immunological status [21, 22]. In the present study, a lower SAS was significantly associated with severe POCs. However, regardless of POCs, a lower SAS was significantly associated with a poor OS. Our findings therefore suggest that the SAS might be a useful prognostic marker either with or without POCs in CRC patients.

The oncological significance of the SAS has been poorly documented in CRC patients. A large amount of intraoperative blood loss and perioperative blood transfusion has been reported to be associated with tumor cell spillage, immunosuppression, and inflammation, thus leading to cancer progression and recurrence [17, 23]. In addition, a poor intravascular blood flow induces the arrest, adhesion, and extravasation of circulating tumor cells preceding metastasis [24]. Furthermore, cancer progression exacerbates the cardiac function [25]. Tumors induce cardiac atrophy and dysfunction through the release of proinflammatory cytokines [26]. In the present study, a lower SAS was significantly associated with an advanced pT, pN, pStage, and blood transfusion before PMS. A lower SAS was significantly associated with a worse RFS and CSS. In particular, a lower SAS was an independent factor for the CSS after PMS. These findings suggest that the SAS might be a biomarker reflecting not only the intraoperative hemodynamics but also cancer progression in CRC patients.

Postoperative adjuvant chemotherapy using doublet therapy of 5-fluorouracil (5-FU) and folic acid (leucovorin, LV) or capecitabine with oxaliplatin (FOLFOX or CapeOX) has been widely considered the standard treatment for patients with stage III CRC after curative resection [27, 28]. However, 20−30% of patients with stage III CRC develop recurrence despite receiving adjuvant chemotherapy [29]. This indicates that there remains room for improvement in the outcomes of such patients. Risk factors for recurrence that can help determine the regimen and duration of adjuvant chemotherapy have not been fully validated. In the present study, a subgroup analysis showed that a lower SAS was significantly associated with a worse OS in patients with pStage II and III CRC. These findings suggest that the SAS might be a prognostic biomarker, regardless of the stage, and may be useful for determining the indication and regimen of adjuvant chemotherapy in CRC patients.

Several limitations associated with the present study warrant mention. First, this study was a retrospective study conducted at a single institution and included patients who underwent both laparoscopic and open surgery, which might have contributed to selection bias. In this study, the PMS minimizes the bias in the clinicopathological characteristics of enrolled patients for internal validation. We need further examination using a public database or other race/ethnicity for external validation. Second, gene mutation, such as BRAF and KRAS mutations, and mismatch repair status, such as microsatellite instability (MSI), were insufficient. Third, data on anesthesia management, such as the volume of infusions, sedatives, and analgesics, were insufficient. Finally, the optimal SAS cutoff value has not yet been determined. The cutoff value in the present study was determined by ROC curve analyses for severe POCs.

## Conclusion

A lower SAS (≤6) was an independent prognostic factor for the OS and CSS after radical surgery in CRC patients. Our results suggest that the SAS might be a useful biomarker predicting oncological outcomes in CRC.

## Data Availability

The datasets generated during and/or analyzed during the current study are not publicly available due to hospital regulations.

## Abbreviations

CRC: Colorectal cancer
POC: Postoperative complication
SAS: Surgical Apgar score
EBL: Estimated blood loss
LMAP: Lowest mean arterial pressure
LHR: Lowest heart rate
OS: Overall survival
RFS: Recurrence-free survival
CSS: Cancer-specific survival
PSM: Propensity score matching
GIST: Gastrointestinal stromal tumor
BMI: Body mass index
GPS: Glasgow prognostic score
CRP: C-reactive protein
ASA-PS: American Society of Anesthesiologists classification of physical status
pT: Pathological T
pN: Pathological N
pStage: Pathological TNM stage
CCI: Charlson comorbidity index
ROC: Receiver operating characteristic
CDC: Clavien-Dindo classification
JSCCR: Japanese Society for Cancer of the Colon and Rectum
CT: Computed tomography
FOLFOX: 5-Fluorouracil/leucovorin plus oxaliplatin
CapeOX: Capecitabine plus oxaliplatin
HR: Hazard ratio
CI: Confidence interval
5-FU: 5-Fluorouracil
LV: Leucovorin
MSI: Microsatellite instability
